# HEARS‐NPS: A Randomized‐Controlled Trial of Community‐Delivered Hearing Care for Persons with Alzheimer’s disease and Related Dementias (ADRD) and their Care Partners

**DOI:** 10.1002/alz70861_108912

**Published:** 2025-12-23

**Authors:** Carrie L Nieman, Peter Hope, Jeannie‐Marie S Leoutsakos, Sevil Yasar, Frank R Lin, George Rebok, Laura N. Gitlin, Kostas G. Lyketsos, Esther S. Oh

**Affiliations:** ^1^ Johns Hopkins University School of Medicine, Baltimore, MD USA; ^2^ Richman Family Precision Medicine Center of Excellence in Alzheimer’s Disease, Johns Hopkins University, Baltimore, MD USA; ^3^ Department of Psychiatry and Behavioral Sciences, Johns Hopkins University School of Medicine, Baltimore, MD USA; ^4^ Division of Geriatric Medicine and Gerontology, Department of Medicine, Johns Hopkins University School of Medicine, Baltimore, MD USA; ^5^ Johns Hopkins Bloomberg School of Public Health, Baltimore, MD USA; ^6^ Johns Hopkins University, Baltimore, MD USA; ^7^ Drexel University, Philadelphia, PA USA; ^8^ Johns Hopkins Bayview Medical Center, Baltimore, MD USA; ^9^ School of Medicine, Johns Hopkins University, and Johns Hopkins Bayview Medical Center, Baltimore, MD USA

## Abstract

**Background:**

Hearing loss is highly prevalent and can have significant consequences for older adults aging with cognitive impairment. Optimizing sensory function may be an important yet overlooked approach to reducing neuropsychiatric symptoms (NPS). Preliminary evidence supports the potential for hearing care interventions to reduce NPS. The Hearing health Equity through Research and Solutions (HEARS) intervention is a theory‐driven hearing care intervention delivered by trained paraprofessionals using over‐the‐counter hearing technology, with demonstrated efficacy among older adults. Previously adapted for older adults with cognitive impairment, the HEARS‐NPS intervention is a refined intervention aims to strengthen communication between care partners and participants with co‐morbid hearing loss and cognitive impairment.

**Method:**

The refined HEARS‐NPS intervention will be evaluated through an NIA‐funded NIA Stage 1b randomized controlled trial with a 6‐week delayed treatment group in a cohort of 150 community‐dwelling participants with cognitive impairment and hearing loss and their care partners.

**Result:**

Dyads will be recruited in partnership with the Johns Hopkins Memory & Alzheimer’s Treatment Center, the Johns Hopkins Alzheimer’s Disease Research Center, and accompanying community outreach. The refined HEARS‐NPS intervention will be delivered virtually by trained interventionists, older adults volunteers, and occur over two structured sessions that consist of 1) fitting and orientation to an OTC device and 2) targeted education and counseling on managing hearing loss in dementia. Outcomes will be assessed at baseline, 6‐weeks, 3‐months, and 6‐months post‐randomization.

**Conclusion:**

The HEARS‐NPS trial seeks to explore the feasibility, acceptability, and preliminary efficacy of an affordable, accessible hearing care intervention as an accompanying non‐pharmacological approach to NPS. The findings from this trial will not only inform best practices in managing NPS but may also be useful in the development of new, cost‐effective non‐pharmacological interventions for individuals with dementia and their care partners.

